# Recommendations for the design of randomized controlled trials in strength and conditioning. Common design and data interpretation

**DOI:** 10.3389/fspor.2022.981836

**Published:** 2022-09-08

**Authors:** Marco Beato

**Affiliations:** School of Health and Sports Sciences, University of Suffolk, Ipswich, United Kingdom

**Keywords:** statistics, randomization, strength training and conditioning, sport, trials, error, evidence

## Introduction

A central aim of strength and conditioning (S&C) coaches is to improve their athletes' performance with exercise prescription (The team physician strength conditioning of athletes for sports: a consensus statement, 2015). Coaches select specific exercises because they have previously had positive experiences with such exercises and because of existing scientific evidence supporting the validity and efficacy of those exercises (Murad et al., 2016; Wackerhage and Schoenfeld, 2021). Research in S&C has drastically increased over the last 20 years, leading to many modern-day practitioners basing their exercise prescription on the most advanced and updated scientific evidence. Therefore, research in S&C plays a key role in the design, implementation, and variation of training protocols (Beato et al., 2021). Sports practitioners, as seen in the field of medicine, have embraced the use of evidence-based practice to improve the likelihood of success (achieving their planned aims) of their training prospection (Wackerhage and Schoenfeld, 2021). However, sport science is plagued by popular beliefs, myths and poor-quality evidence (Gabbett and Blanch, 2019). There are many reasons why the quality of articles is sometimes low, for example, the authors' knowledge of research methods or statistics is inadequate (Cleather et al., 2021; Sainani et al., 2021), the resources invested in the research are limited, or the research was carried out in a hurry, which could be related to many reasons, for instance, several studies are performed by students who have limited time and experience when performing data recording (Abt et al., 2022). Consequently, a key question remains: what should we do to improve current scientific evidence and limit the spread of new low-quality evidence? While it is assumed that not all published articles are of high quality and that in some cases there may be errors (Sainani et al., 2021), this should not be common (Smith, 2006). Consequently, the objective of this article is to make some recommendations in the field of research design, specifically, randomized controlled trials (RCTs) and data interpretation, with the aim of improving the robustness of future S&C research (e.g., training, performance, injury prevention) and avoiding the replication of common mistakes.

## Evidence pyramid

S&C prescription should be based on the most relevant and updated scientific evidence, as reported above, following where possible an evidence-based approach (Murad et al., 2016; Wackerhage and Schoenfeld, 2021). Researchers and practitioners who design training protocols should be aware of the evidence pyramid (Murad et al., 2016), where evidence is categorized based on robustness (derived from study type). At the bottom of the pyramid, we find experts' opinions and case reports, while at the top we find meta-analyses and systematic reviews, followed by (a level lower) RCTs. Practitioners should design their training protocols using the evidence on the top of this pyramid and if such evidence is missing, they can use the less robust articles up to the last level. If there is no solid evidence, expert opinions can be useful. However, such opinions should be considered for what they are and should not be assumed as true – in particular when they are based on unpublished data or on exclusively personal arguments. Despite this, researchers and coaches should work together to verify the validity and effectiveness of strategies that coaches are already using based on their experience gained with athletes.

In the field of S&C, which is the main focus of this article, we are well aware that some of the most common limitations are the length of interventions (frequently too short) (Rothwell, 2006), a low number of participants enrolled (the calculation of the sample power is also frequently missing), and the lack of a control group in the study design (Moher et al., 2001). With such limitations in mind, the effect of the intervention is often influenced by other factors not associated with the protocol that can undermine the evidence's robustness. Therefore, it is important that researchers avoid these errors and increase the robustness of their intervention studies [also embracing open-science (Calin-Jageman and Cumming, 2019)] to provide stronger evidence to practitioners, who can apply such evidence later in their daily practice.

## Recommendations for the design of RCTs

There is the need for more robust evidence and the design of RCTs (following CONSORT guidelines) (Moher et al., 2001) should be a priority for researchers in the S&C field in order to verify training interventions. Researchers and practitioners can find some recommendations for the design of RCTs in the following lines (see Table 1).

**Table 1:** Summary of the recommendations for the design of randomized controlled trials and data interpretation in strength and conditioning.

**Evidence pyramid**	**Recommendations for the design of RCTs**	**Errors and sample size**	**Other considerations**
The central aim of S&C coaches is to improve their athletes' performance with exercise prescription. S&C prescription should be based on the most relevant and updated scientific evidence following where possible an evidence-based approach.	Following CONSORT guidelines and learn from clinical medicine (see phases of RCTs). Sport science practitioners should design small-sample studies when weak evidence exists, but to avoid designing many of them where some robust evidence already exists and to increase the sample size of their studies to answer different research questions.	Type I error: a training method is found effective when it is actually ineffective (false positive). Type II error: a training method is found ineffective when it is actually effective (false negative).	A common statistical error is the use of within-group comparisons instead of between-group comparisons to determine longitudinal differences between interventions. The experimental and control groups must be directly compared.
Evidence pyramid categorized evidence based on robustness. At the top of the pyramid, we find meta-analyses and systematic reviews, followed by (a level lower) RCTs, while at the bottom we find experts' opinions and case reports.	Researchers need to control for bias and confounding factors. Researchers and practitioners can use different types of randomization such as simple, block, stratified, unequal randomization, and covariate adaptive randomization.	Inadequate sample size: small samples, first, increase the chance of making a type II error, second such underpowered studies could struggle to find difference between interventions (or a control group) spreading wrong evidence, third they could be a waste of time and money for researchers and athletes.	This paper uses null hypothesis significance testing for assessing differences between interventions, but significance testing/*p*-values answer a very narrow question and should never be the sole focus.
Practitioners should design their training protocols using the evidence on the top of this pyramid and if such evidence is missing, they can use the less robust articles up to the last level.	Practitioners should be aware of the differences that exist between designs such as RCTs, superiority and non-inferiority trials, and they should select the most adequate research design based on the existing evidence reported in the literature.	Practitioners should be aware that the sample size matters, and adequately powered studies should be prioritized because they offer more robust evidence. Practitioners could use G*Power to calculate the statistical power of their studies (Figure 1).	*P*-values are often used dichotomously (yes or no decision-making process). Researchers also need to consider the effect sizes and CIs.
Common limitations in the field of S&C are the length of interventions (too short), a low number of participants enrolled, and the lack of a control group in the study design.	Control group: Researchers can involve a traditional control group (no-intervention group), or they can compare the effect of a new intervention vs. active control, specifically, an intervention which has been proven to be effective (e.g., current best-practice treatment).	CIs are related to the selection of the alpha value, CIs = (1 - alpha value)*100%. Therefore, an alpha value of 5% corresponds to a 95% CIs. Using 90% CIs is possible, but this decision should be justified because using it increases the risk of Type I error.	CIs provide critical information beyond statistical significance such as they provide a plausible range of values for the true effect and reveal the precision of the estimate.

RCTs, Randomized controlled trials; S&C, Strength and conditioning; CONSORT, Consolidated Standards of Reporting Trials; CIs, Confidence intervals.

### Conducting all four phases of RCTs

To enhance our research design knowledge, researchers in S&C could learn something from clinical medicine (Atkinson et al., 2008). Clinical trials are classified into phases based on the objectives of the trial. Phase 1 trials are the first studies that verify the effect of an intervention and are usually carried out involving small samples (e.g., larger single-group or controlled study) (Evans, 2010), phase 2 trials involve a larger sample (e.g., RCTs) and aim to understand, for instance, the efficacy of an intervention vs. a control or the dose-response relationship, phase 3 trials should aim to confirm the efficacy of an intervention using a larger sample (e.g., collaboration between research groups), while phase 4 trials are “confirmatory or registration” trials (Atkinson et al., 2008; Evans, 2010). If we try to transfer what has just been said to S&C, despite the differences between medicine/clinic and sport, we can understand that the size of the selected sample of a trial (and phase) should be based on the existing level of knowledge on the subject. Therefore, if a new training method is to be studied, small sample sizes may be adequate, but if this training method has already been proven effective, it would not be adequate to continue to carry out small studies, instead future trials should involve large samples (e.g., evaluating dose-response relationship).

Sport scientists could consider the design of a framework of this type in the future; however, it is not suggested here that sport scientists must label their trials in phases. Instead, this study recommended to design small-sample studies when weak evidence exists (subsequently, these studies can be combined in a meta-analysis), but to avoid designing many of them where some robust evidence already exists and to increase the sample size of their studies to answer different research questions, for instance, confirm the efficacy of an intervention with a lower training dose or compare different interventions (e.g., superiority trials).

Practitioners and researchers should also be aware of practical problems associated with the design of RCTs. RCTs are sometimes impractical in sport research for various reasons such as not enough athletes in the elite team to split into two groups, unwillingness of the coach to have a parallel control group, and logistical difficulties with having two kinds of training. In this case, other designs (e.g., repeated-measures) aimed at overcoming these problems can be a valid alternative (Vandenbogaerde et al., 2012), although they have a lower position (therefore robustness) in the evidence pyramid.

### Randomization

RCTs are studies that aim to verify a research hypothesis in which a number of participants (e.g., athletes) are randomly assigned to some groups that correspond to some specific training protocols. A simple example of an RCT could be the comparison between an innovative resistance training method vs. a control group or an active control based on the existing knowledge (current best-practice treatment as control). To successfully verify that this innovative resistance training method is effective (alternative hypothesis) (Calin-Jageman and Cumming, 2019), researchers need to control for bias and confounding factors (Evans, 2010). Randomization is a way to control for such factors, therefore, the participants of the groups should be randomly allocated into these groups and not arbitrarily selected by the researchers (or coaches). In such a case, we should speak about a non-randomized controlled trial, which is a different study design with lower robustness (Sedgwick, 2014). Researchers and practitioners can use different types of randomization such as simple, block, stratified, unequal randomization (a smaller randomization ratio such as a ratio of 2:1), and covariate adaptive randomization (Suresh, 2011).

### Control group and active control

A key step for the robustness of an RCT is the selection of a control group. A control group, in particular for phase 1 and 2 studies, should avoid performing any relevant training protocol which could affect the validity of the trial. It is clear that if researchers do not know the efficacy of a new training method, they need to verify its effect vs. a control group. When some RCTs with these characteristics have been successfully performed, researchers could state that this new method is effective (if enough RCTs are available, a meta-analysis could be performed) (Liberati et al., 2009). In sport and medicine there is an alternative to RCTs using a no-intervention control group, that is a trial that involves an active control. This situation is very frequent in sport because many training methods are known to be effective, therefore designing trials with a no-intervention group (as control) that involve athletes is sometimes impractical or considered unethical. In this case, the researchers do not involve a no-intervention group, but they compare the effect of a new intervention vs. an intervention which has been proven to be effective (e.g., current best-practice treatment). In clinic, this approach is used when a standard of care treatment already exists, therefore the new treatment should be tested against it and, for example, proven to be superior (i.e., superiority trials or non-inferiority trials) (Schiller et al., 2012).

Considering what was reported above, researchers in S&C should be aware of the differences that exist between trials of different phases and between designs such as RCTs, superiority and non-inferiority trials, and they should select the most adequate research design (to answer their research question) based on the existing evidence reported in the literature.

## Common mistakes that we can find in RCTs

Some of the common mistakes that can be found in RCTs are: the selection of inadequate sample size, the use of an inadequate alpha level, the use of flawed statistical methods, and the wrong interpretation of the results of the study.

### Inadequate sample size

**Figure 1:**
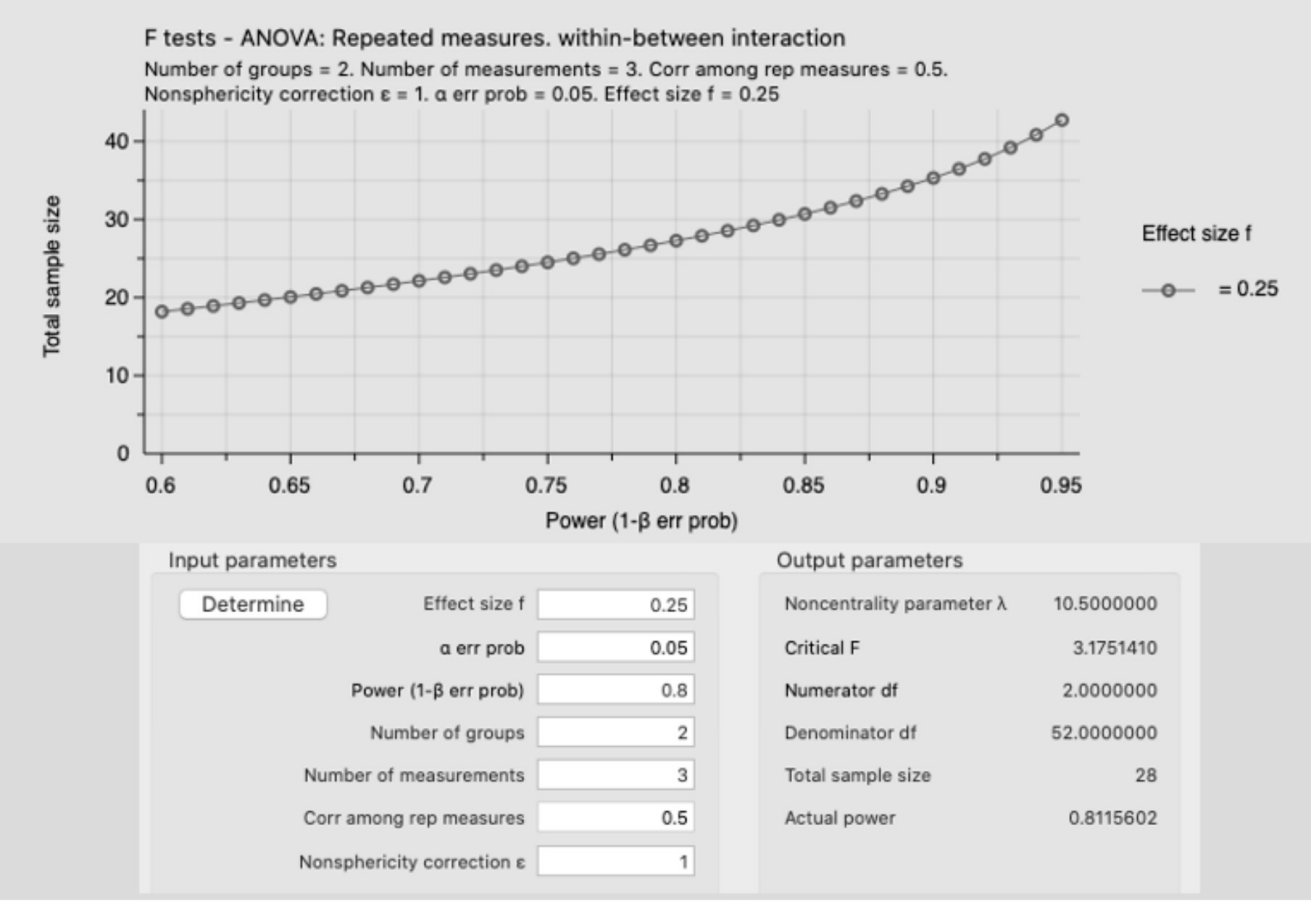
Example of an a priori power analysis using an ANOVA, repeated measures, within-between interaction with a *medium* effect size (f = 0.25) and an alpha error prob of 0.05 (5%). The total sample size for this study is 28 participants, with an actual power of 0.811. Moreover, this figure shows that increasing the sample size (y axis) is possible to increase the power (1-beta err prob), for instance, recruiting 35 participants would increase the sample power to 0.9, which would decrease the type II error.

The use of inadequate sample size is a limitation that has been reported in several methodological papers and it should not surprise anyone with research experience (Sainani and Chamari, 2022), however, the design of underpowered studies is still very common in S&C (Beck, 2013). Researchers and practitioners should be aware that small samples, first, increase the chance of making a type II error, which means that a training method is found ineffective when it is actually effective (false negative) (Evans, 2010), second such underpowered studies could struggle to find difference between interventions (or a control group) spreading wrong evidence, third they could be a waste of time and money for researchers and athletes (Atkinson and Nevill, 2001a). Therefore, practitioners and researchers should be aware that the sample size matters, and adequately powered studies should be prioritized because they offer more robust evidence. Researchers and practitioners could use G^*^Power (which is a free-to-use software) to calculate the statistical power of their studies (as reported in the example in Figure 1). Further information about G^*^Power can be found here: https://www.psychologie.hhu.de/arbeitsgruppen/allgemeine-psychologie-und-arbeitspsychologie/gpower.

### Type I error and confidence intervals

Type II error is an issue; however, it is more “dangerous” to design intervention studies using an unsuitable alpha level, which can lead to the claim that an intervention is effective when it is not (false positive) (Evans, 2010). In S&C as well as in clinic (or medicine) the most common alpha level is 5% (*p* = 0.05) (Peterson and Foley, 2021). CIs are related to the selection of the alpha value, CIs = [1 - alpha value]^*^100% (Chow and Zheng, 2019), therefore an alpha value of 5% corresponds to a 95% CIs. Researchers in S&C should be well-aware of the differences of using either 95% CIs or 90% CIs because the type I error would be affected. Previous researchers reported that it is unethical to use lower alpha (or a one-tailed test) just to show that a difference is significant, and therefore the decision on the use of 90% CIs should be justified (in advance, e.g., pre-registration) (Atkinson and Nevill, 2001b). It is important to clarify that researchers can use the alpha level they considered more suitable for their research if this is properly justified but they should not use 90% CIs as default (it increases risk of false positive). A clear example of this issue is reported by Diong (2019) in a letter to the editor related to the paper published by Pamboris et al. (2019), where is reported that the use of a 90%CIs are more likely to report an effect that does not exist (type I error). A final consideration concerns the use of a one-tailed test, which has more probability to find a difference between the groups (e.g., intervention vs. control), but this test should be used if the researchers want to determine if there is a significant difference in one direction, while there is no interest in verifying if a difference in the other direction exists.

### Data interpretation and statistical methods

Another important issue is the use of flawed statistical methods; one example in sport science is the use of magnitude-based inference (or magnitude-based decision analyses), which is a controversial statistical approach that has never been adopted by the statistical community (Sainani, 2018; Sainani et al., 2019). Although this approach has been used in hundreds of papers in sport science, it has been repeatedly been demonstrated as unsound and it should not be used in S&C (or sport science) research (Sainani, 2018; Lohse et al., 2020). Magnitude-based inference reduces the type II error rate (false negative) but with the tradeoff is a much higher type I error rate (Sainani, 2018). This method has also been labeled as Bayesian, but it is not universally accepted to actually be Bayesian (Welsh and Knight, 2015).

This paper uses null hypothesis significance testing for assessing differences between interventions; however, significance testing/ *p*-values answer a very narrow question, *p-*values are often used dichotomously (yes or no decision-making process, e.g., *p* = 0.049 or *p* = 0.051, respectively) (Betensky, 2019), therefore, they should never be the sole focus; researchers also need to consider the effect sizes and CIs.

### Between-group and within-group comparisons

Another common statistical error is the use of within-group comparisons instead of between-group comparisons to determine longitudinal differences between interventions. Many researchers (or practitioners) conclude that an intervention is successful if there is a significant within-group difference in the experimental group but not in the control group or if the effect size of the experimental group is larger than the effect size of the control group. However, this is not the correct comparison (Nieuwenhuis et al., 2011)–the experimental and control groups must be directly compared, for instance with ANOVA or ANCOVA.

### Interpretation of the results based on confidence intervals–when it does cross zero

It is common to find papers that use CIs to make decisions but interpret them incorrectly. Since there is a one-to-one-correspondence between CIs and *p-*values (as explained above), this means that if the CI about a between-group mean difference (e.g., in an RCT) does not cross zero, there is a statistically significant difference between the intervention and the control group, while if the CI does cross zero, it means that there is no significant difference between groups at the specific alpha value selected (e.g., 5% that corresponds to 95% CIs). However, there are still cases where researchers wrongly interpret CIs (Diong, 2019; Mansournia and Altman, 2019). For instance, in this paper (Pamboris et al., 2019), some CIs of between-condition comparisons crossed zero, meaning that there is not a statistically significant difference between conditions, yet the authors still claimed to find a difference (as subsequently explained in this letter, Diong, 2019). Importantly, CIs provide critical information beyond statistical significance such as they provide a plausible range of values for the true effect and reveal the precision of the estimate (Sainani, 2011).

## Conclusion

This paper aimed to make some recommendations in the field of research design and data interpretation with the aim of improving the robustness of future S&C research and avoiding the replication of common mistakes that can be found in the sports literature. Much can be learned from the clinical field therefore practitioners, coaches and researchers should be encouraged to adopt research methods coming from such research area when they design RCTs. In S&C there is the need for more robust RCTs which should have longer duration, greater number of participants enrolled, and the right type of control group (no-intervention control or active control) based on the existing knowledge. Finally, researchers should be aware of some common mistakes that should be avoided such as the selection of a sample of inadequate dimension (type II errors) or inadequate alpha levels (risk of type I), the use of flawed statistical methods, and the incorrect selection of a statistical test or the wrong interpretation of CIs of their study.

## Author contributions

The author confirms being the sole contributor of this work and has approved it for publication.

## Conflict of interest

The author declares that the research was conducted in the absence of any commercial or financial relationships that could be construed as a potential conflict of interest.

## Publisher's note

All claims expressed in this article are solely those of the authors and do not necessarily represent those of their affiliated organizations, or those of the publisher, the editors and the reviewers. Any product that may be evaluated in this article, or claim that may be made by its manufacturer, is not guaranteed or endorsed by the publisher.
